# Supplementary material for: Nanoelectrode design from microminiaturized honeycomb monolith with ultrathin and stiff nanoscaffold for high-energy micro-supercapacitors
Source: Nat Commun. 2020 Jan 15;11:299. doi: 10.1038/s41467-019-14170-6 (PMC6962208; doi:10.1038/s41467-019-14170-6)
Supplement: Supplementary file 1 — Supplementary Information [file 41467_2019_14170_MOESM1_ESM.pdf]

Supplementary Information for

Nanoelectrode design from microminiaturized honeycomb monolith with ultrathin and stiff nanoscaffold for high-energy micro-supercapacitors

Lei et al.

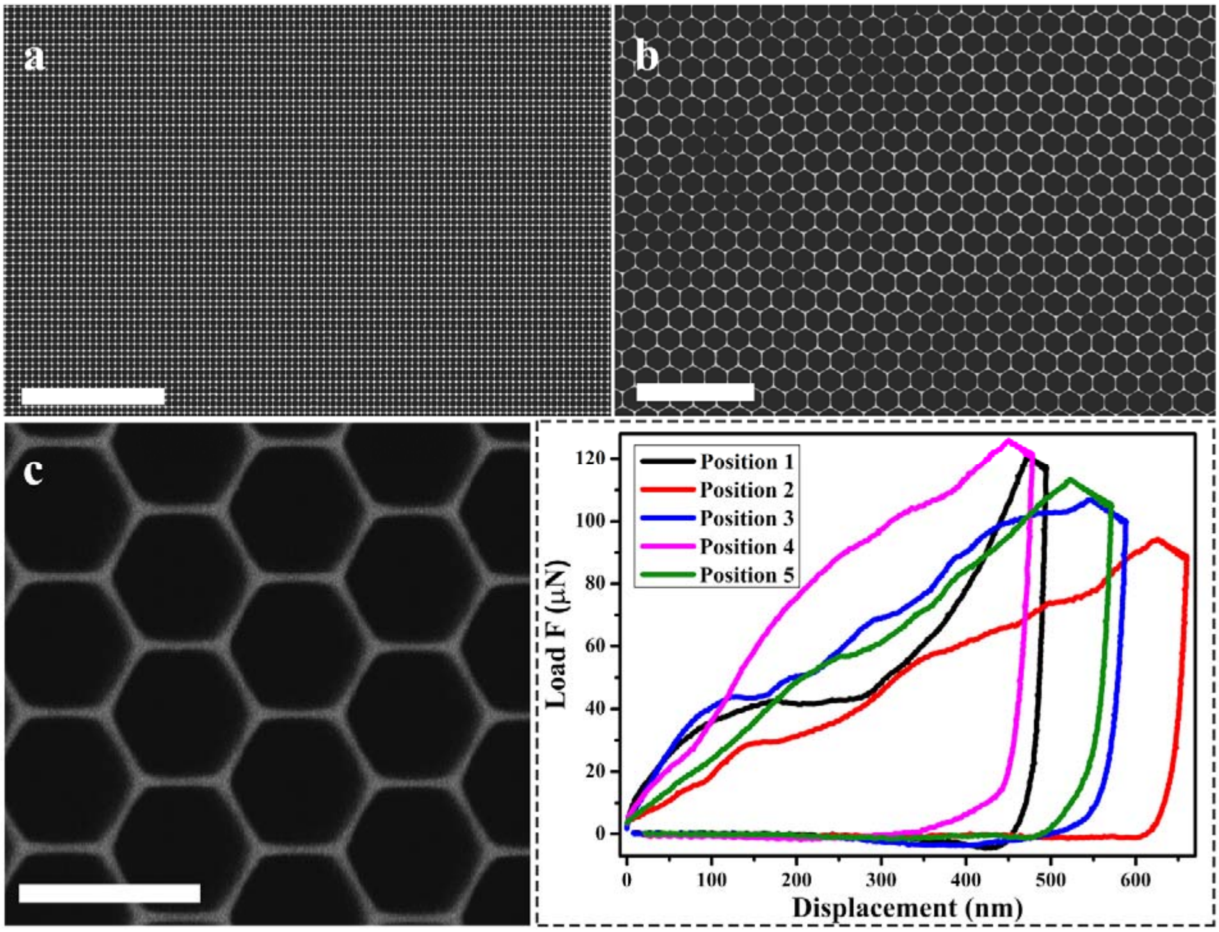

Supplementary Figure 1 | Morphology and mechanical properties of HAN. a Large-scale SEM image of HAN with square cell arrangement and 400 nm inter-cell spacing. **b** Large-scale SEM image of HAN with hexagonal cell arrangement and 800 nm inter-cell spacing. **c** The load-displacement (L-D) curves at the different positions of the HAN (hexagonal cell arrangement and 400 nm inter-cell spacing) with an indentation load of 150 μN at room temperature. The Young's modulus of the HAN obtained from L-D curves are 3.22, 1.46, 1.50, 2.05 and 1.41 GPa at the positions 1 – 5, respectively. Scale bar: 8 μm (**a**); 4 μm (**b**); 500 nm (**c**)

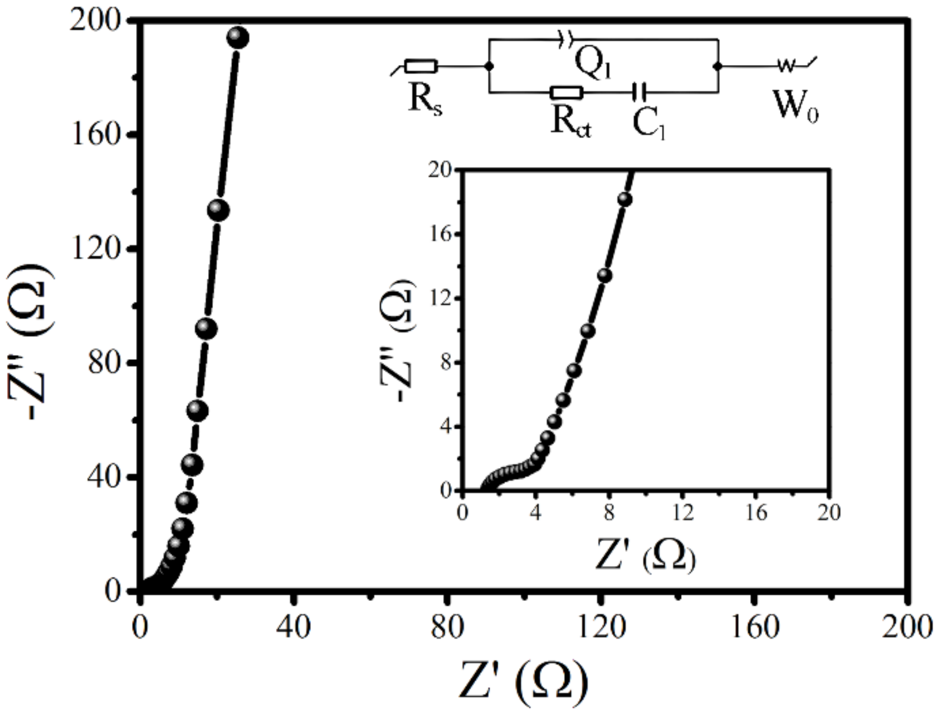

Supplementary Figure 2 | Electrochemical impedance of HAN@SnO$_2$. The Nyquist plots of HAN@SnO$_2$//HAN@SnO$_2$ device.

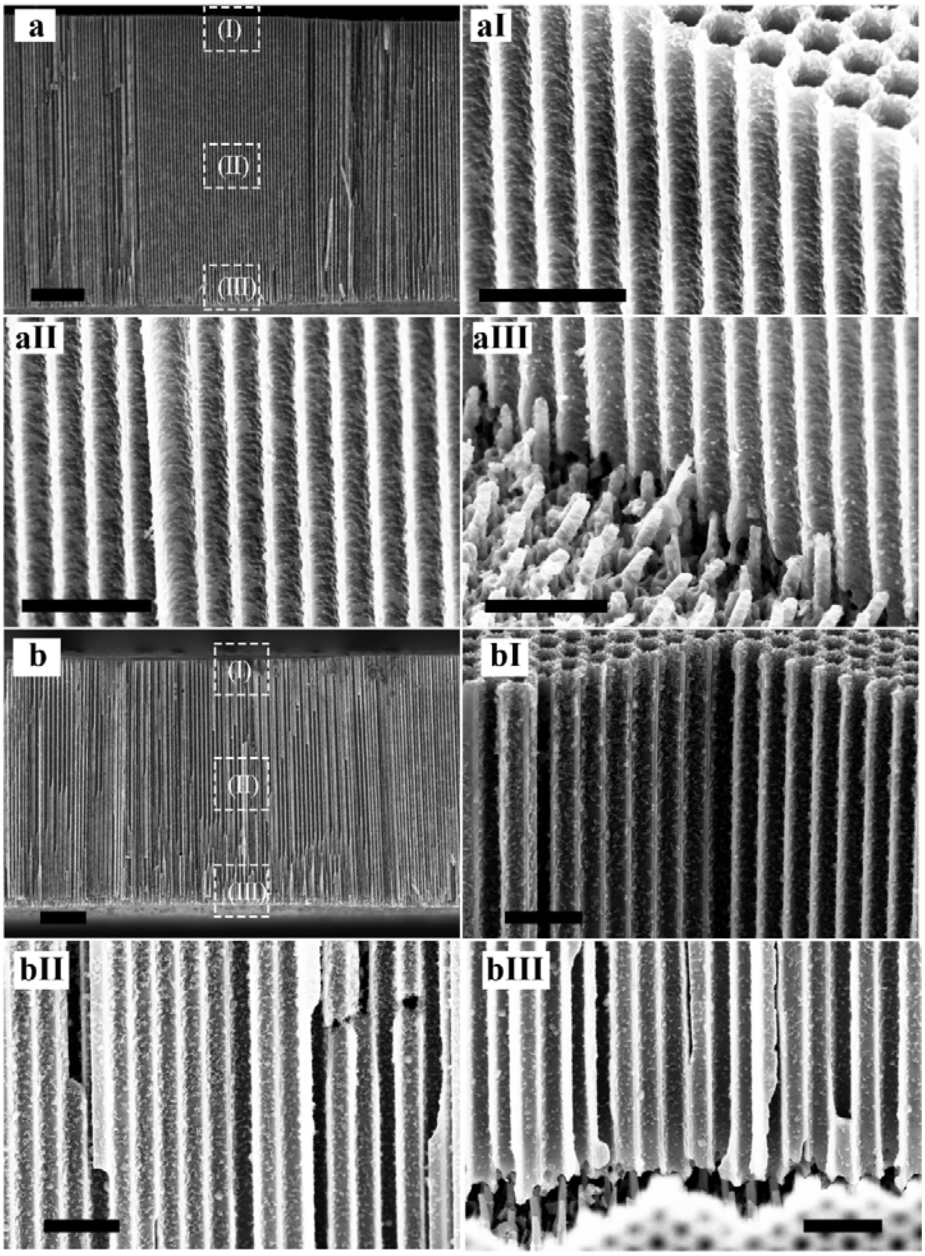

Supplementary Figure 3 | Cross-sectional SEM images of HAN@SnO_2@PPy and HAN@SnO_2@MnO_2 electrodes with a HAN cell depth of 25 μm. a Overall view of HAN@SnO_2@PPy electrode and corresponding high-resolution SEM image recorded from the region marked in **aI**, **aII** and **aIII**. **b** Overall view of HAN@SnO_2@MnO_2 electrode and corresponding high-resolution SEM image recorded from the region marked in **bI**, **bII** and **bIII**. Scale bar: 5 μm (**a**); 1 μm (**aI**); 1 μm (**aII**); 1 μm (**aIII**); 5 μm (**b**); 1 μm (**bI**); 1 μm (**bII**); 1 μm (**bIII**)

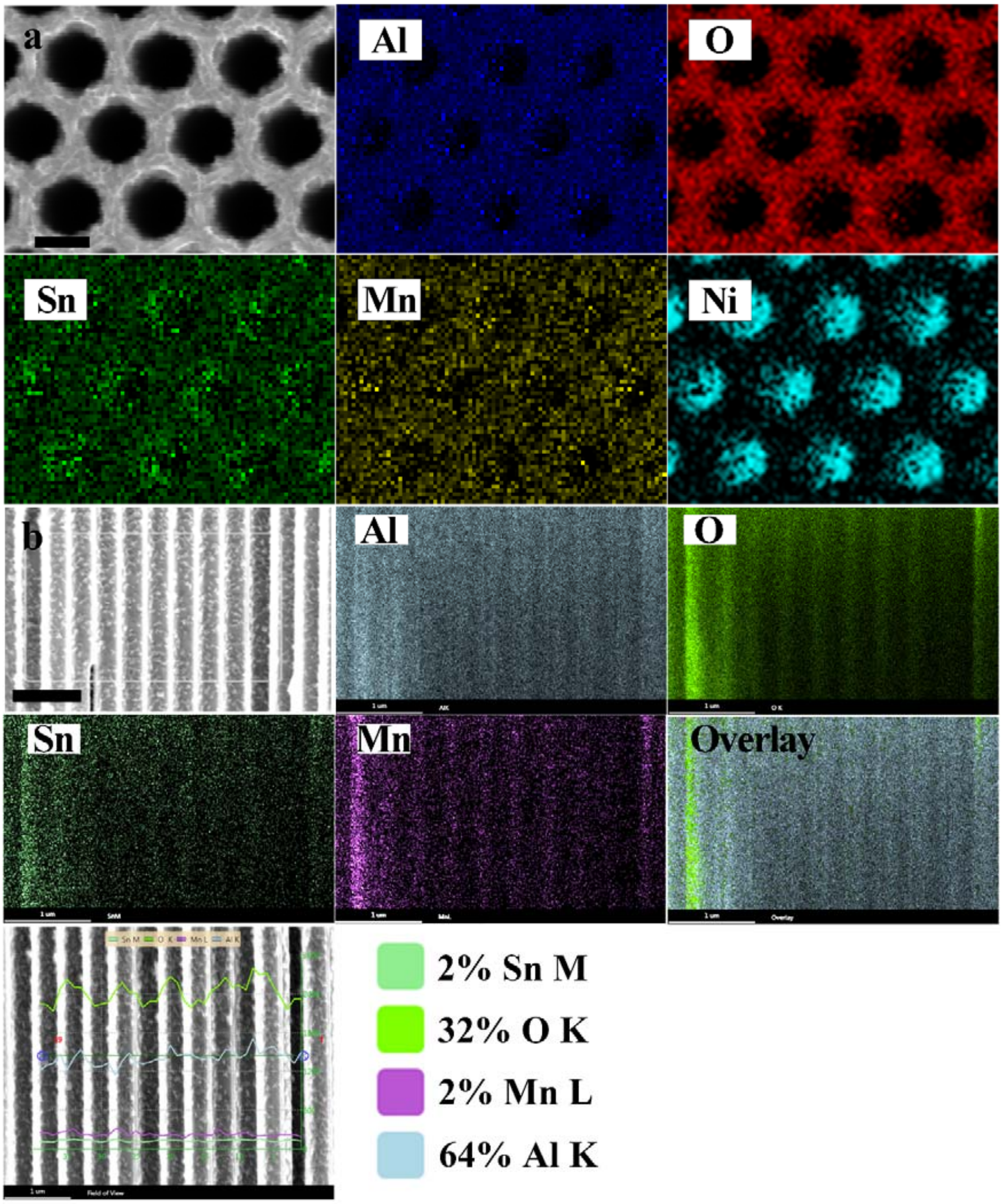

Supplementary Figure 4 | Top-view and cross-sectional EDX mapping of HAN@SnO_2@MnO_2. a Top-view and **b** cross-sectional view. Scale bar: 250 nm (**a**); 1 µm (**b**)

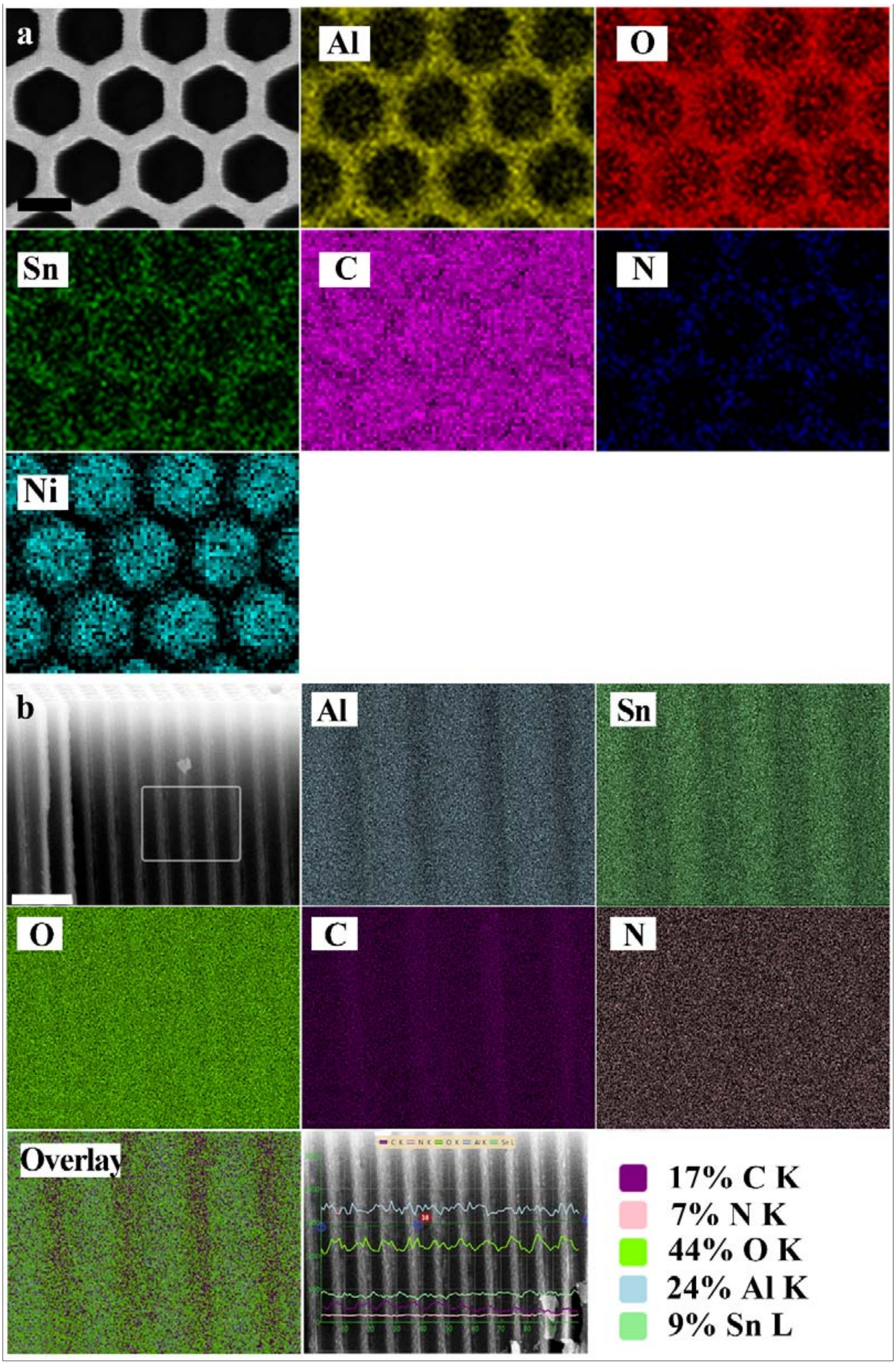

Supplementary Figure 5 | EDX mapping of HAN@SnO_2@PPy. a Top-view and **b** cross-sectional view.

Scale bar: 250 nm (**a**); 500 nm (**b**)

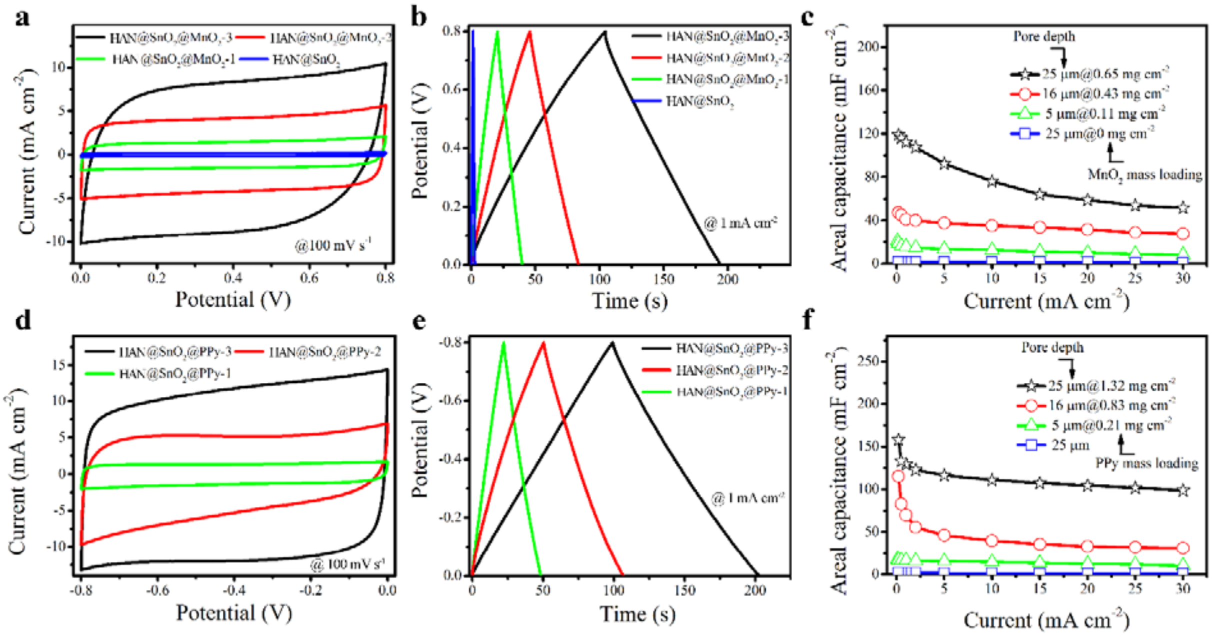

Supplementary Figure 6 | Electrochemical performance of the symmetric stacked MSCs based on HAN-reinforced pesudocapacitive nanoelectrodes. **a** The CV curves, **b** GCD profiles and **c** device areal capacitance as a function of current densities of HAN@SnO_2@MnO_2//HAN@SnO_2@MnO_2 MSCs with different pore thickness. **d** The CV curves, **e** GCD profiles and **f** device areal capacitance as a function of current densities of HAN@SnO_2@PPy//HAN@SnO_2@PPy MSCs with different HAN cell depth. The electrolyte is 1.0 M Na_2SO_4 aqueous solution.

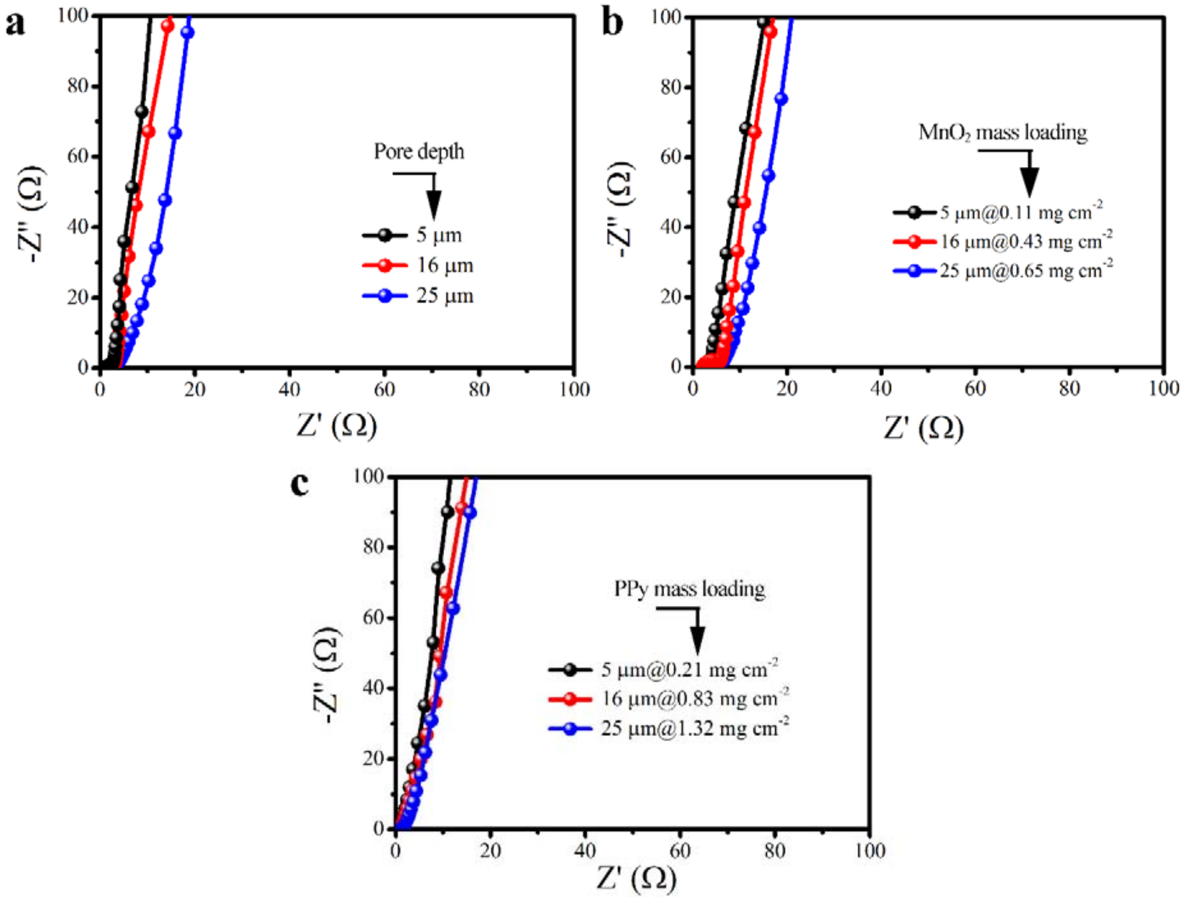

Supplementary Figure 7 | Electrochemical impedance properties comparison of symmetric MSCs. Warburg region of **a** HAN@SnO_2, **b** HAN@SnO_2@MnO_2, and **c** HAN@SnO_2@PPy electrodes with different pore depth of HAN. The electrolyte is 1.0 M Na_2SO_4 aqueous solution.

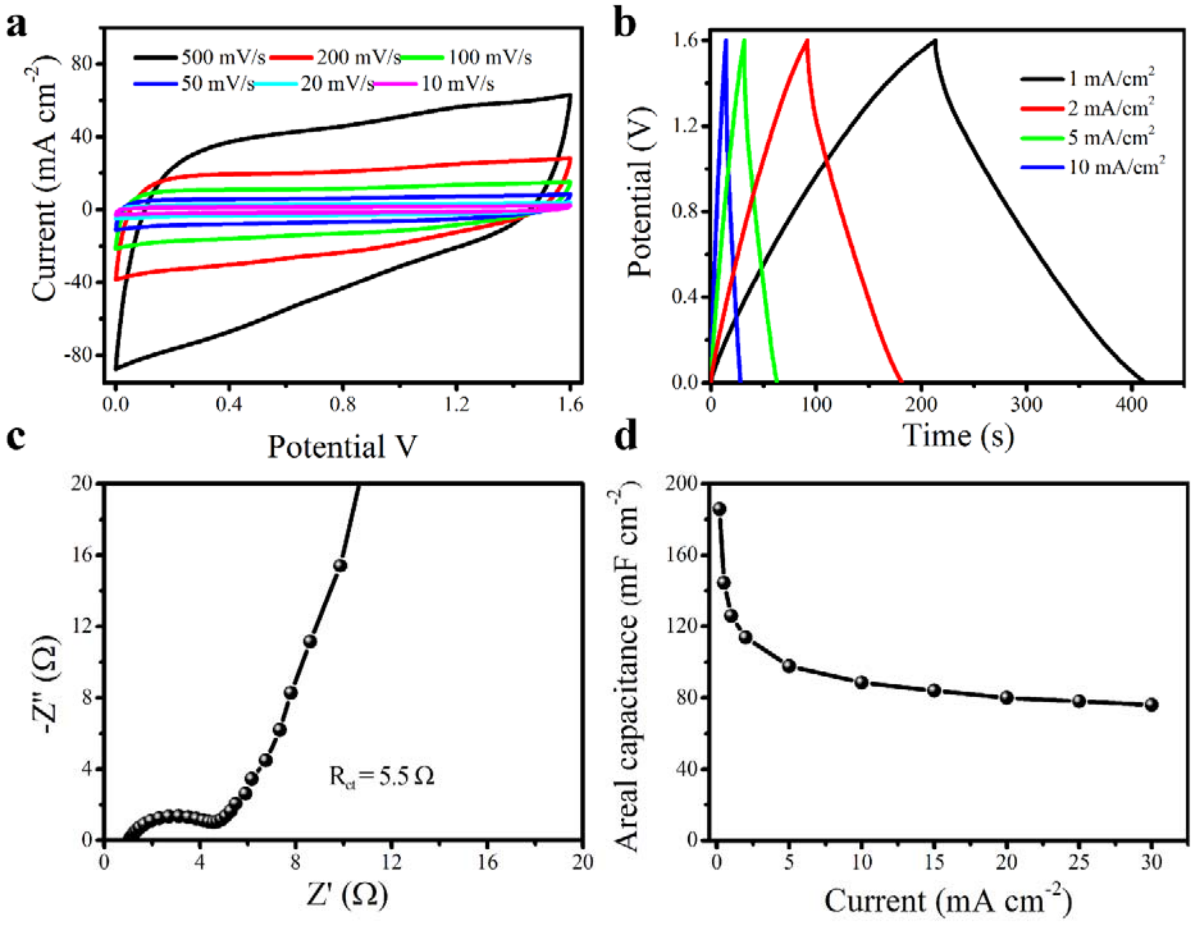

Supplementary Figure 8 | Electrochemical performance of HAN@SnO_2@MnO_2//HAN@SnO_2@PPy asymmetric stacked MSCs. a The CV curves, **b** GCD profiles, **c** Nyquist plots and **d** device areal capacitance as a function of current densities of HAN@SnO_2@MnO_2//HAN@SnO_2@PPy MSCs (For asymmetric MSCs based on positive and negative electrodes are both with 25-µm-deep cell). The electrolyte is 1.0 M Na_2SO_4 aqueous solution.

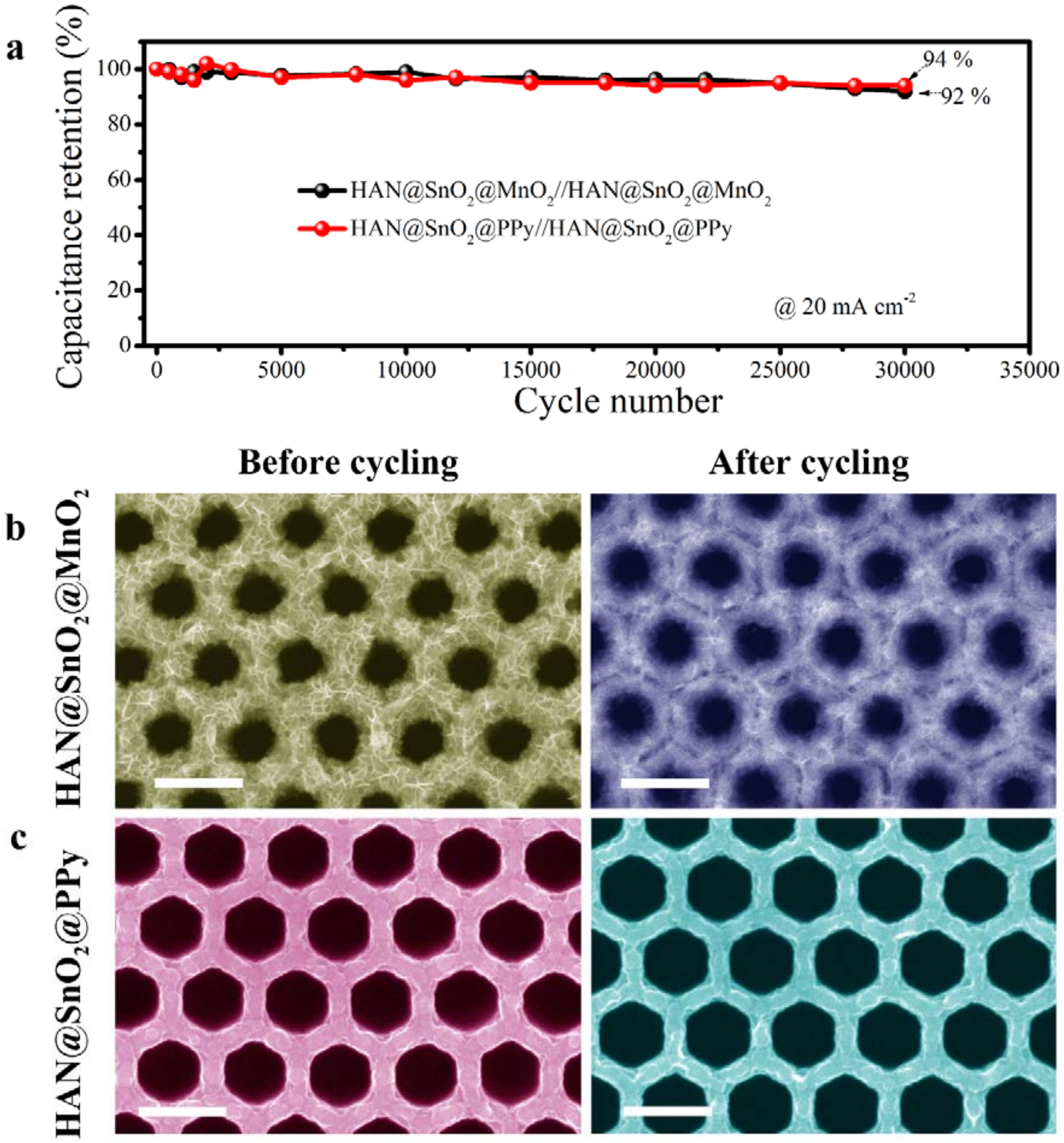

Supplementary Figure 9 | Cyclic performance of symmetric MSCs and morphological stability of HAN-based nanoelectrodes. a Cycling ability of HAN@SnO_2@MnO_2//HAN@SnO_2@MnO_2 symmetric MSCs and HAN@SnO_2@PPy//HAN@SnO_2@PPy symmetric MSCs, respectively, measured at a current density of 20 mA cm^{-2} for 30,000 continued charge-discharge cycles. SEM images of **b** HAN@SnO_2@MnO_2 and **c** HAN@SnO_2@PPy electrodes before (left) and after (right) 30,000 cycles. Scale bar: 400 nm (**b**, **c**)

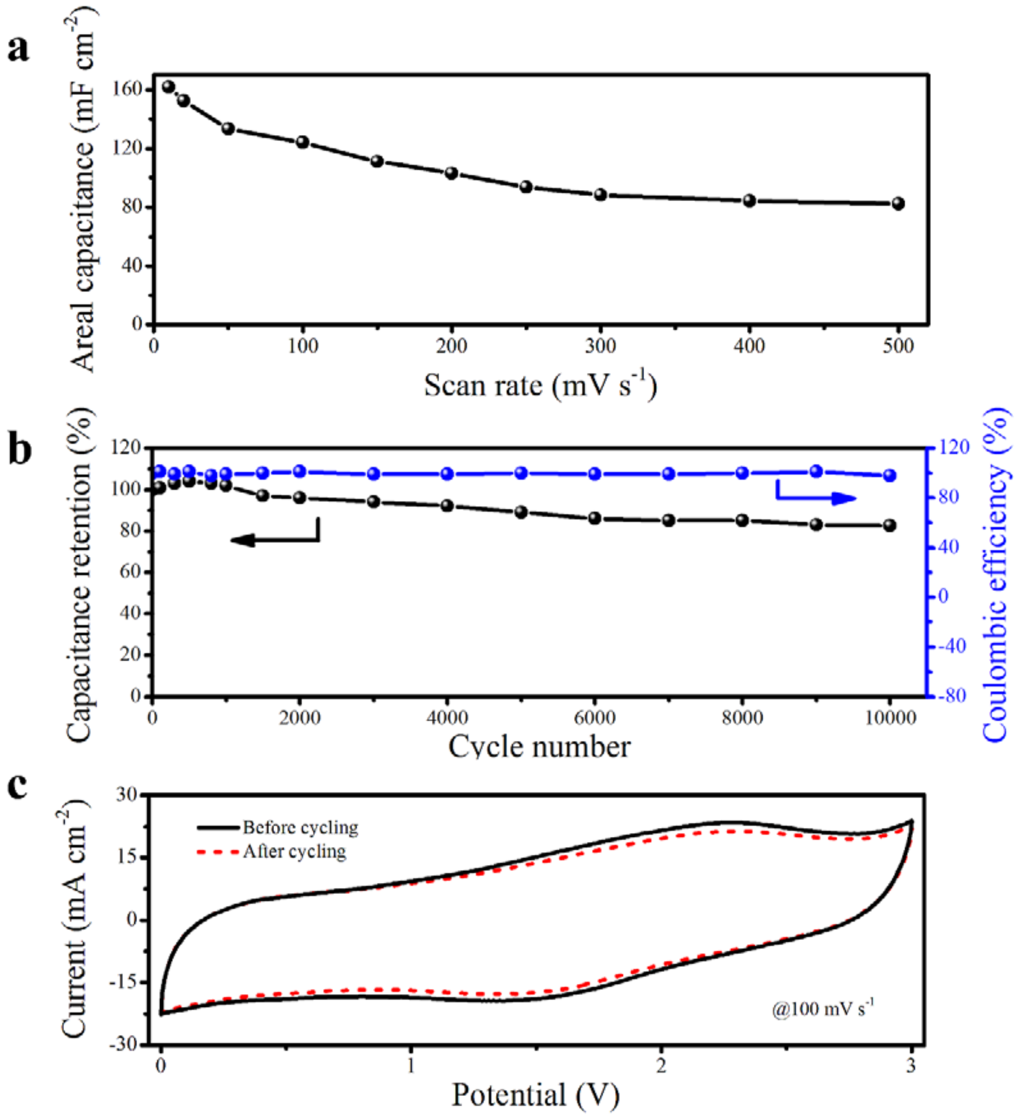

Supplementary Figure 10 | Device capacity of HAN@SnO_2@MnO_2//HAN@SnO_2@PPy asymmetric stacked MSCs with EMIM-TFSI electrolyte. a Device areal capacitance as a function of scan rates of HAN@SnO_2@MnO_2//HAN@SnO_2@PPy MSCs. **b** Cycling stability test at 20 mA cm^{-2} for 10000 times and **c** corresponding CV curves at 100 mV s^{-1} before and after cycling. The positive and negative electrodes are both with 25-μm-deep HAN.

Supplementary Table 1 | Performances of state-of-the-art MSCs. Device performance of HAN@SnO_2@MnO_2//HAN@SnO_2@PPy (abbreviated as MnO_2//PPy) in comparison with various MSCs reported recently.

Electrode material	Electrode thickness (μm)	Electrolyte	Potential window (V)	Device capacitance (mF cm^{-2})	Device energy (μWh cm^{-2})	Device power (mW cm^{-2})	Cycling stability	C_{1000}/C_0 (%)	*Ref.*
Graphene	0.7	PSSH	1.0	<1	<1	<1	11,000	~100	[1]
PG	2.0	$BMIMPF_6$	3.0	<1	<1	<1	2,000	~100	[2]
CNTCs	20	BMIM-BF_4	3.0	6	3.6	69	8,000	~100	[3]
ACF	1.5	1 M H_2SO_4	0.6	<1	<1	-	10,000	~100	[4]
OLC	7	1 M Et_4NBF_4 in PC	3.0	<1	<1	177	10,000	~100	[5]
AC	5	0.5 M H_2SO_4	3.0	<1	<1	20	10,000	~100	[5]
LWG	20	1.0 M TEABF4	1.0	2.5	<1	-	10,000	~100	[6]
Graphene	25	1 M H_2SO_4	1.0	4	<1	9	9,000	~98	[7]
PEDOT	2.4	1 M H_2SO_4	0.8	18.7	2	0.2	1,000	~90	[8]
CDC	4.1	2 M $EMIBF_4$ in AN	3.0	41.8	40	30	11,000	~100	[9]
LSG/MnO_2	15	1 M Na_2SO_4	0.9	385	60	-	10,000	100	[10]
MnO_2//Bi_2O_3	-	1 M Na_2SO_4	1.8	97	43.4	12.9	4,000	90	[11]
MnO_2//PPY	1.8	1 M Na_2SO_4	1.7	~30	~10	0.5	2,000	90	[12]

NN@MnO_2	5	1 M Na_2SO_4	0.8	~100	20	10	5,000	~90	[13]
Zn NSs	50	2 M $ZnSO_4$	1.5	~324	115	0.16	10,000	~100	[14]
rGO/IL/CP	0.3	PVA-H_3PO_4	3.0	27.4	32	1.05	-	-	[15]
MXene	-	PVA-H_2SO_4	0.5	43	0.32	0.15	14,000	~97	[16]
MHCF	~0.18	PVA-LiCl	1.8	19.8	9.49	-	5,000	96.8	[17]
MXene	3.2	$EMIMBF_4$/PVDF-HFP	3.0	42	13.9	4.5	10,000	~100	[18]
Y-$Ti_3C_2T_x$	~0.1	PVA/H_2SO_4	0.6	61	0.63	0.33	10,000	93.7	[19]
WJM-G-SWCNTs	~23	PVA-H_3PO_4	1.8	1.32	0.064	20	10,000	~100	[20]
H-SiC	~16	PVA-KCl	0.8	23.6	5.2	11.2	10,000	94	[21]
$Cu(OH)_2$@FeOOH	14	$EMIMBF_4$	1.5	58	18	-	10,000	80	[22]
3D RuO_2	46	0.5 M H_2SO_4	0.9	-	91.4	-	2,000	~100	[23]
PDMS/GF	260	0.5 M Na_2SO_4	1.6	592	106	16	12,000	~100	[24]
Ni-CAT MOF	~200	PVA-LiCl	1.4	15.2	4.1	7	5,000	87	[25]
MnO_2-ITO NWs	28	1 M Na_2SO_4	1.0	194	27	15	20,000	61	[26]
**MnO_2//PPy**	**25 µm**	**1 M Na_2SO_4**	**1.6**	**186**	**66**	**24**	**30,000**	**~100**	**This work**
**MnO_2//PPy**	**25 µm**	**EMIM-TFSI**	**3.0**	**128**	**160**	**40**	**10,000**	**~100**	

References

1. Li, J., et al. Scalable Fabrication and Integration of Graphene Microsupercapacitors through Full Inkjet Printing. *ACS Nano* **11**, 8249-8256 (2017).
2. Xiao, H., et al. One-step device fabrication of phosphorene and graphene interdigital micro-supercapacitors with high energy density. *ACS Nano* **11**, 7284-7292 (2017).
3. Lin, J., et al. 3-dimensional graphene carbon nanotube carpet-based microsupercapacitors with high electrochemical performance. *Nano Lett.* **13**, 72-78 (2012).
4. Wei, L., Nitta, N. & Yushin, G. Lithographically patterned thin activated carbon films as a new technology platform for on-chip devices. *ACS Nano* **7**, 6498-6506 (2013).
5. Pech, D., et al. Ultrahigh-power micrometre-sized supercapacitors based on onion-like carbon. *Nat. Nanotechnol.* **5**, 651-654 (2010).
6. Gao, W., et al. Direct laser writing of micro-supercapacitors on hydrated graphite oxide films. *Nat. Nanotechnol.* **6**, 496–500 (2011).
7. Lin, J., et al. Laser-induced porous graphene films from commercial polymers. *Nat. Commun.* **5**, 5714 (2014).
8. Kurra, N., Hota, M. K. & Alshareef, H. N. Conducting polymer micro-supercapacitors for flexible energy storage and AC line-filtering. *Nano Energy* **13**, 500-508 (2015).
9. Huang, P., et al. On-chip and freestanding elastic carbon films for micro-supercapacitors. *Science* **351**, 691-695 (2016).
10. El-Kady, M. F., et al. Engineering three-dimensional hybrid supercapacitors and microsupercapacitors for high-performance integrated energy storage. *Proc. Natl. Acad.*

Sci. U.S.A. **112**, 4233-4238 (2015).

11. Xu, H., Hu, X., Yang, H., Sun, Y., Hu, C. & Huang, Y. Flexible asymmetric micro-supercapacitors based on Bi_2O_3 and MnO_2 nanoflowers: larger areal mass promises higher energy density. *Adv. Energy Mater.* **5**, 1401882 (2015).
12. Grote, F. & Lei, Y. A complete three-dimensionally nanostructured asymmetric supercapacitor with high operating voltage window based on PPy and MnO_2. *Nano Energy* **10**, 63-70 (2014).
13. Liu, L., Zhao, H., Wang, Y., Fang, Y., Xie, J. & Lei, Y. Evaluating the role of nanostructured current collectors in energy storage capability of supercapacitor electrodes with thick electroactive materials layer. *Adv. Funct. Mater.* **28**, 1705107 (2018).
14. Zhang, P., et al. Zn-ion hybrid micro-supercapacitors with ultrahigh areal energy density and long-term durability. *Adv. Mater.* **31**, 1806005 (2018).
15. Gao, J., et al. Laser-assisted multiscale fabrication of configuration-editable supercapacitors with high energy density. *ACS Nano* **13**, 7463-7470 (2019).
16. Zhang, C. J., et al. Additive-free MXene inks and direct printing of micro-supercapacitors. *Nat. Commun.* **10**, 1795 (2019).
17. He, Y., et al. Nano-sandwiched metal hexacyanoferrate/graphene hybrid thin films for in-plane asymmetric micro-supercapacitors with ultrahigh energy density. *Mater. Horiz.* **6**, 1041-1049 (2019).
18. Zheng, S., et al. Ionic liquid pre-intercalated MXene films for ionogel-based flexible micro-supercapacitors with high volumetric energy density. *J. Mater. Chem. A* **7**,

9478-9485 (2019).

19. Zhang, C., et al. Stamping of flexible, coplanar micro-supercapacitors using MXene inks. *Adv. Funct. Mater.* **28**, 1705506 (2018).

20. Bellani, S., et al. Scalable poduction of graphene inks via wet-jet milling exfoliation for screen-printed micro-supercapacitors. *Adv. Funct. Mater.* **29**, 1807659 (2019).

21. Li, W., et al. All-solid-state on-chip supercapacitors based on free-standing 4H-SiC nanowire arrays. *Adv. Energy Mater.* **9**, 1900073 (2019).

22. Xie, J.-Q., et al. In situ growth of $Cu(OH)_2$@FeOOH nanotube arrays on catalytically deposited Cu current collector patterns for high-performance flexible in-plane micro-sized energy storage devices. *Energy Environ. Sci.* **12**, 194-205 (2019).

23. Ferris, A., Bourrier, D., Garbarino, S., Guay, D. & Pech, D. 3D Interdigitated microsupercapacitors with record areal cell capacitance. *Small* **15**, 1901224 (2019).

24. Wang, Y., et al. Monolithic integration of all-in-one supercapacitor for 3D electronics. *Adv. Energy Mater.* **9**, 1900037 (2019).

25. Wu, H., Zhang, W., Kandambeth, S., Shekhah, O., Eddaoudi, M. & Alshareef, H. N. Conductive metal–organic frameworks selectively grown on laser-scribed graphene for electrochemical microsupercapacitors. *Adv. Energy Mater.* **9**, 1900482 (2019).

26. Du, J., et al. High-performance pseudocapacitive microsupercapacitors with three-dimensional current collector of vertical ITO nanowire arrays. *J. Mater. Chem. A* **7**, 6220-6227 (2019).
